# Can Teenage Men Be Targeted to Prevent Teenage Pregnancy? A Feasibility Cluster Randomised Controlled Intervention Trial in Schools

**DOI:** 10.1007/s11121-018-0928-z

**Published:** 2018-07-18

**Authors:** Maria Lohan, Áine Aventin, Mike Clarke, Rhonda M. Curran, Clíona McDowell, Ashley Agus, Lisa McDaid, Chris Bonell, Honor Young

**Affiliations:** 1 School of Nursing and Midwifery, Queen’s University of Belfast, Medical Biology Centre, 97 Lisburn Road, Belfast, BT9 7BL UK; 2 Centre for Public Health, Institute of Clinical Sciences, Block B, Queen’s University Belfast, Royal Victoria Hospital, Belfast, BT12 6BA UK; 3 Northern Ireland Clinical Trials Unit, Elliott Dynes Building, Royal Hospitals, 274 Grosvenor Road, Belfast, BT12 6BA UK; 4 MRC/CSO Social & Public Health Sciences Unit, University of Glasgow, Top floor, 200, Renfield Street, Glasgow, G2 3QB UK; 5 Department of Social and Environmental Health Research, London School of Hygiene & Tropical Medicine, Keppel Street, London, WC1E 7HT UK; 6 School of Social Sciences, Cardiff University, Glamorgan Building, King Edward VII, Cardiff, CF10 3WT UK

**Keywords:** Sex education, Gender, Teenage pregnancy, Intervention, Feasibility trial

## Abstract

The World Health Organisation advocates a direct focus on adolescent men in reducing adolescent pregnancy; however, no trials have been conducted. This trial (ISRCTN11632300; NCT02092480) determines whether a novel Relationship and Sexuality Educational intervention, *If I Were Jack*, is acceptable and feasible to implement in mixed sex UK classrooms. The intervention is a teacher-delivered intervention that emphasises male alongside female  responsibility in preventing unintended pregnancies and is designed to prevent unprotected sex. The trial was a parallel-group cluster randomised controlled feasibility trial with embedded process and cost evaluation in eight secondary schools (unit of randomisation) among 831 pupils (mean age 14) in Northern Ireland, alongside a qualitative evaluation of transferability in ten schools in Scotland, Wales and England. The sampling strategy was a maximum variation quota sample designed to capture a range of school management types. Four schools were randomised to each arm and the control arm continued with usual practice. Study duration was 12 months (from November 2014), with follow-up 9 months post-baseline. Results demonstrated that the intervention was acceptable to schools, pupils and teachers, and could be feasibly implemented, cost-effectively, with minor enhancements. The between-group difference in incidence of unprotected sex (primary outcome at pupil level) of 1.3% (95% CI 0.5–2.2) by 9 months demonstrated a potential effect size consistent with those reported to have had meaningful impact on teenage pregnancy. The study responds to global health policy for a paradigm shift towards inclusion of men in the achievement of sexual and reproductive health goals in a practical way by demonstrating that a gender-sensitive as well as a gender transformative intervention targeting males to prevent teenage pregnancy is acceptable to adolescent men and women and implementable in formal education structures. *If I Were Jack* now merits further effectiveness testing.

**Electronic supplementary material:**

The online version of this article (10.1007/s11121-018-0928-z) contains supplementary material, which is available to authorized users.

## Introduction

### Why Men?

Feminist research has transformed health science in general, such that it is no longer scientifically acceptable to model either the causes or the cures of ill-health without taking sex and, in more progressive research, the gender continuum into account (Connell 2012). Yet, in the field of reproductive health research, the field arguably most driven through feminist concerns, men are often overlooked (Lohan 2015). Not only is there a need for a paradigm shift in the gender bias in reproductive health research, but there is a need for a shift in the practical interventions that are intended to achieve an improvement in reproductive health goals, such as a reduction in teenage pregnancies worldwide.

The latest systematic reviews of educational interventions targeted to the prevention of teenage pregnancies speak to a number of innovative approaches, including combining school- and community-based approaches; combining education, skills building and contraception promotion; targeted programmes to at risk groups or areas; and incentive-based interventions to maintain school attendance (Chin et al. 2012; Mason-Jones et al. 2016; Oringanje et al. 2016; Goesling et al. 2017). While teenage men are rarely excluded from these innovations designed to address teenage pregnancy, equally, they are seldom the central focus (for exceptions, see Gruchow and Brown 2011; Herrman et al. 2016; Pulerwitz et al. 2015). Yet, it is acknowledged at the global health policy level that tackling the gendered power relationships in society that relinquish some men from reproductive/contraceptive responsibility (arguably more prevalent in liberal high-income nations) while giving some men power over women’s reproductive bodies, such as their right to access contraceptive and pregnancy termination services (perhaps more prevalent in conservative low-income countries), is a substantial part of the problem in addressing teenage pregnancy world-wide (United Nations 2015; WHO 2017; WHO 2011).

### Why This Intervention?

We report the results of a cluster randomised controlled feasibility trial (cRCT) of a schools-based Relationship and Sexuality Educational (RSE) intervention, which especially emphasises the role of teenage men in preventing teenage pregnancy and is delivered to both males and females aged 14–16. The intervention, *If I Were Jack*, which is centred around an interactive video drama with accompanying classroom materials and activities is described more fully below. This is the first intervention to be developed and trialled which explicitly promotes a gender-sensitive approach to addressing teenage pregnancy by focussing on male perspectives and a gender transformative perspective by encouraging males to share reproductive responsibility. It is also the first controlled trial of an RSE intervention in the UK that reports the recruitment of faith-based schools.

The complex socio-economic determinants of unintended pregnancy have been précised in systematic reviews (Acharya et al. 2010; Harden et al. 2009; Imamura et al. 2007; Lohan et al. 2010) which highlight that while RSE on its own may have limited effects in reducing the incidence of teenage pregnancy, it has played an important role in its decline in recent decades (Mason-Jones et al. 2016; Pound et al. 2016; MacDowall et al. 2015; Swann et al. 2003). Furthermore, high-quality RSE is considered fundamental to respecting and fulfilling the right of the child to appropriate sexual health education (United Nations 2015).

We report the results of a feasibility trial and additional transferability study designed to rigorously test acceptability and feasibility of implementation of the intervention. The trial was conducted in Northern Ireland and the qualitative transferability study was conducted in the other countries of the UK. The results of this paper inform the literature on whether or not there is demand for a new approach to education on preventing teenage pregnancy which explicitly focuses on males; the possible reach of this intervention into a diversity of schools; the cost of delivery; and whether or not a future effectiveness trial is possible and warranted. In addition, the reported study prepares for a future effectiveness trial and is in line with the UK Medical Research Council’s (2006) recommendations for a staged approach to the evaluation of complex interventions. In line with the standards for feasibility studies outlined by Bowen et al. (2009), the following outcome measures are reported in this paper: demand, acceptability, implementation/practicality (including cost of delivery), integration and preliminary effectiveness of the primary outcome that we will use in a future effectiveness trial, namely unprotected sex. We use the CONSORT guideline for cRCTs to describe the method (Campbell et al. 2012) complemented by the Template for Intervention Description and Replication (TIDieR) checklist (Hoffmann et al. 2014) to describe the intervention. We first report methods for the feasibility trial and then the methods for the transferability study. The latter was designed according to the principles of Wang et al. (2005).

## Method

### Trial Design

A pragmatic unblinded parallel-group (1:1 concealed allocation) cluster randomised controlled feasibility trial (cRCT) with embedded process and economic evaluations.

### Participants and Recruitment

All registered secondary schools in Northern Ireland (NI) were eligible for inclusion with the exception of eight schools with fewer than 30 pupils aged 14. In order to test feasibility in a broad spectrum of schools and robustness of trial methods for a future effectiveness trial, eight schools were sought for recruitment during 4 months of 2014 using a maximum variation sampling method capturing school management types to include:

1. Secondary schools (managed by the state) in deprived areas; these schools tend to have a higher intake of pupils of protestant religion.
2. Grammar Schools (managed by the state) which select pupils by academic selection and have higher rates of pupils from high socio-economic status households, and a higher intake of protestant pupils.
3. Roman Catholic (RC) schools (managed by state and RC church), including grammar and secondary with the distinctions noted above and have a higher intake of RC pupils.
4. All other types of schools, including integrated religious state-managed schools.

Schools were approached at RSE training events, through introduction by members of the trial steering and advisory groups or by letters of invitation. Eligible participants were all pupils (along with subject teachers) in year 4 of participating secondary schools (mean age 14.45 years at baseline) with the exception of those who were unable to read the questionnaire. Following a decision to participate by school principals, schools sent letters and a DVD informing parents about the study. Pupils were provided with information sheets by teachers and an overview of the study by the research team.

### Randomisation and Blinding

Clusters, the unit of randomisation, were secondary schools in NI. Randomisation was conducted following baseline data collection by the NI Clinical Trials Unit (NICTU), a UK Clinical Research Collaboration (UKCRC) registered CTU. Schools were randomised as pairs stratified by their school management type using simple randomisation (via the computer software NQuery 3.0), to ensure intervention and control arms contained similar types of variation of schools. Although concealed allocation was deployed (by NICTU, who were not involved in recruitment) at the cluster level, blinding was not possible because this trial, pragmatic by nature, was designed to evaluate a teacher-led intervention to change pupil behaviour in a real-world secondary school classroom setting. Schools in the control group continued with usual RSE practice, whilst schools in the intervention group received the 4-week *If I Were Jack* intervention in replacement of usual RSE practice. Schools were retained in the study for a period of 12 months (November 2014 to November 2015), with follow-up 1 occurring at 5 months and follow-up 2 at 9 months post-baseline.

**Table:** 

Intervention	
Name and brief description: *If I Were Jack* is an evidenced-based educational resource designed to prevent unintended pregnancy by increasing teenagers’ intentions to avoid unprotected sexual intercourse. It is especially designed to provoke thought on the role of teenage men in preventing unintended teenage pregnacy, but is designed to engage both males and females in mixed-sex settings. In development since 2013, it has involved sustained communication with target groups, including pupils, teachers and parents, along with a wide range of stakeholders. A detailed description of the design process, including logic model, is available (Aventin et al. 2015).	
*Why, rationale of essential elements: If I Were Jack* is built on an innovative set of concepts which previous research has identified as contributors to effective RSE education. These include: • Activities targeting theoretically informed behavioural and psycho-social correlates of risk behaviour including knowledge, attitudes, perceptions of risk, peer and gender norms, self-efficacy in communication and intention to avoid sexual risk-taking behaviour (Swann et al. 2003; Gavin et al. 2010) • Engagement of teenagers by addressing the operation of gender and social class norms, age-appropriateness, cultural relevance and integration of interactive media (Bailey et al. 2010; Gavin et al. 2010) • Opportunities for pro-social peer communication, communication with parents/guardians (Swann et al. 2003; Gavin et al. 2010) • Teacher training in relation to the intervention and teenage pregnancy (Swann et al. 2003; Gavin et al. 2010)	
*What, a description of the materials:* (i) The *If I Were Jack* opening interactive video drama (IVD) is a culturally sensitive (locally filmed in NI) film intended to immerse teenagers in a story of a week in the life of Jack, a teenager who has just been told his girlfriend is pregnant. By asking males and females to imagine they were Jack and how they would think and feel if they were in his situation, it is designed to expose and challenge the gender assumptions around roles and responsibilities for teenage pregnancy by opening them up for reflection and negotiation. (ii) Classroom materials for teachers with four detailed lesson plans with specific classroom-based and homework activities which provide pupils with sexual health information and opportunities for discussion, skills practice, reflection and anticipatory thinking. (iii) Sixty-minute face-to-face training session for teachers provided by the researcher. (iv) Sixty-minute information/discussion session for parents/guardians led by RSE teachers. (v) Information brochures and factsheets about the intervention and unintended teenage pregnancy for schools, teachers, teacher trainers, young people and parents.	
*Who delivers*? Trained RSE teachers to pupils, incorporating significant peer discussion.	
*How, modes of delivery*: To be delivered during four consecutive RSE lessons in classroom settings. The IVD is to be delivered on individual computers/tablets with headphones.	
*Where, locations where intervention has occurred*: In NI and Ireland, using a further locally produced IVD for Ireland. A version of the IVD has been delivered in South Australia.	

### Outcome Measures

#### Demand

The demand for the intervention was assessed through willingness of both intervention and control schools to participate in the trial without the use of incentives. We stipulated that we should recruit at least 25% of schools approached, no more than 25% of parents should withdraw their children from the study and no more than 25% of pupils should refuse to participate.

#### Acceptability

Quantitative measures of acceptability related to retention of both control and intervention schools. We stipulated that no more than one school should withdraw from the study. Qualitative measures of acceptability were an analysis of the satisfaction of pupils and teachers with regard to the intervention and teachers’ willingness to use the intervention in the future, along with independent observations of engagement and reactions during one lesson in each school in the intervention group.

#### Implementation/Practicality, Integration and Economic Cost

Implementation and practicality were assessed through adherence to delivery by teachers and participation by pupils as intended in the intervention group. Teachers completed an implementation log and discussed delivery at interview, and the research team independently observed one intervention session in each school. Implementation and practicality was also assessed with pupils through process evaluation focus group interviews.

The economic costs of the intervention were calculated in the intervention group, using the principle of opportunity cost, to measure the resources actually used in the delivery of each component of the intervention in terms of time input and materials, and a qualitative analysis of the teaching activities displaced or time gained as a result of the intervention being implemented. Regarding the use of actual resources, a micro-costing approach was taken from a public sector decision maker perspective, guided by Ritzwoller et al. (2009). Relevant costs were identified, measured and valued in monetary units using the 2013/2014 price year. Two cost stages were defined. Stage one referred to recurring costs associated with printing the classroom materials for teachers, providing the IVD and the delivery of teacher training. These costs were recorded by the research team prospectively via a specifically designed Teacher Resource Use Questionnaire. Stage 2 referred to the teacher time input for lesson preparation, printing or photocopying of worksheets and teaching, and administration time input for printing or photocopying of worksheets. Pre-start-up costs associated with the development of intervention were not included in the analysis because these are non-recurring costs incurred prior to the start of this study. The trainer in the trial was a researcher employed by the study team, and the costs of this training are included, but no costs were associated with the training location as training took place at schools. Data collection on displaced teaching activities was conducted by interviews with teachers and analysed as part of process evaluation.

#### Preliminary Study of Effects

As this was a feasibility trial, it was not powered to detect effectiveness of the intervention. The purpose of the analysis was to produce an effect-size estimation for a larger effectiveness trial. The primary intended effect associated with this intervention is avoidance of unprotected sex. We evaluated this using a composite primary outcome measure to include self-reports of abstinence from sexual intercourse (delay initiation of sex or return to abstinence) or avoidance of unprotected sexual intercourse at time of last sex. This was determined by use of a questionnaire administered to individual participating pupils (available online). While a reduction of teenage conceptions may be regarded as the ideal, objective primary outcome measure due to bias introduced in self-report measures (Mason-Jones et al. 2016), the sample size in an effectiveness trial would be prohibitively large to have sufficient power to detect change in teenage conceptions. In addition, teenage conception rates are not collected in NI health and social care data, prohibiting the use of data linkage to collect these data for this trial. Unprotected sex during teenage years, measured through self-reports of last sexual intercourse and ever had sexual intercourse, is well established as the main proximate behavioural determinant of teenage pregnancy (Henderson et al. 2010; Stephenson et al. 2003), and studies indicate that, although other behavioural determinants (such as frequency of sexual intercourse and number of sexual partners) are important, avoidance of unprotected sex via consistent use of contraception is central in explaining variation in levels of teenage pregnancy (Swann et al. 2003; Santelli et al. 2007).

### Procedure

#### Questionnaire Data Collection

Participating pupils were asked to complete a paper questionnaire that had previously been piloted. They did this under exam conditions overseen by a researcher during an RSE lesson at baseline (prior to randomisation) and at 5- and 9-month follow-up. A fieldworker was present during data collection to administer and retrieve anonymised questionnaires. Teachers stood at the top of the class only. Additional blank questionnaires were provided for teachers to administer to absent pupils to complete shortly thereafter.

#### Statistical Methods

Primary analyses related to the outcome variables of recruitment, retention and analysis of preliminary effects. Preliminary effect was analysed on an intention to treat basis, using all participants with complete data for baseline and follow-up; in the groups, they were randomised to, regardless of the extent of their engagement with *If I Were Jack* in the case of the intervention arm. Differences between the trial groups were reported using descriptive statistics, generated in STATA. Effect sizes were determined from the differences in proportions between the intervention and control arms, and no statistical analysis to account for clustering was made at this stage.

#### Qualitative Data Collection and Analysis

Data were collected by the primary researcher with the following triangulated data sources in intervention schools to assess acceptability and implementation.

##### Pupils

Following intervention, focus group interviews were conducted across the four intervention schools, two in each of three schools and one in the fourth school (seven groups, *n* = 39). Teachers were asked to choose from volunteers, guided by the principal of selecting a cross section of the class (e.g. sex and academic ability) and inclusion of friendship pairs, to assist focus-group dynamics. Focus groups were held in the school at lunch time, convened by the researcher only, and lasted up to 60 min.

##### Senior School Staff

Semi-structured interviews (*n* = 6) were conducted with School Principals/Vice Principals or Heads of Year on completion of intervention.

##### Teachers

Focus group (four groups, *n* = 10) and semi-structured interviews (*n* = 3) were conducted with RSE teachers in intervention schools. These usually lasted 90 min. Teacher resource use and implementation questionnaires were also collected in each school and discussed at interview. Four further interviews were conducted with teachers in each of the control schools to assess current RSE provision.

##### Researchers

Field notes were obtained from observations of one lesson in each intervention school and during data collection.

Data were analysed using thematic analysis (Braun and Clarke 2006) whereby data were inductively and deductively coded and organised using NVivo to reflect related categories and overarching themes. In relation to focus group interviews, attention was also paid to group dynamics (Hyde et al. 2005). In order to ensure methodological rigour and reliability of both the coding and analysis process, one researcher coded all data and a second independently coded a subset of transcripts and resolved discrepancies through discussion.

### Qualitative Transferability Study

A convenience sample of ten schools was recruited with purposeful inclusion, where possible, of schools in high and low areas of deprivation and to include faith based schools. Three schools were in North and South Wales, four schools in central Scotland and three in the Greater London area of England (Table 1). Two of the schools were faith-based schools (one in England and one in Wales). Researchers approached schools directly by telephone and email as well as accessing schools through school research networks in England and Wales to explain the rationale of the study. Following consent procedures, data were collected with pupils through focus group interviews in each school (*n* = 10). These interviews occurred in classroom settings in small groups (ranging from two to eight participants for eight focus groups), while two group interviews occurred with the whole class. The researcher showed an excerpt of the IVD and shared examples of the classroom exercises at the outset of the interview and interviews lasted between 40 and 80 min. Data were also gathered from RSE teachers/pastoral care teachers or healthy school co-ordinators in nine of the ten schools either through a combination of small focus group interviews (*n* = 5) or individual interviews (*n* = 6). Data were analysed according to the principles of qualitative research outlined above, at first by the team of researchers in each site, and then collectively.

**Table 1:** Schools recruited to transferability study

School ID	Country	Location	School management type
1	England	London (urban)	Academy Converter Mainstream
2		London (urban)	Academy Sponsor Led (Church of England)
3		London (urban)	Academy Converter Mainstream
4	Scotland	East Central (urban)	State
5		East Central (semi-urban)	State
6		West Central (urban)	State
7		West Central (urban)	State
8	Wales	South Wales (urban)	Roman Catholic
9		West Wales (rural)	State
10		North Wales (rural)	State

## Results

We report the results for the feasibility trial, process and cost evaluations in relation to specified outcome measures and how the transferability study complements these results.

### Demand

Within the feasibility trial, 21 schools were approached, 20 responded (95% response rate) and eight schools in NI were successfully recruited as proposed, giving a recruitment rate of 38% (the target being 25%) within the recruitment period of 4 months. Demand was also demonstrated by the most common reasons offered by schools to participate, namely that the intervention would be useful to pupils and that the intervention had strong credibility with teachers. The study demonstrated demand across the full range of the most common types of schools in NI. We successfully recruited schools of the differing school management types, including faith-based schools, schools which are academically high achieving (grammar schools) and those which are less academically focused (secondary schools), and schools in economically deprived as well as economically affluent catchment areas calculated by reference to percentage of pupils eligible for free school meals (Table 2). Nonetheless, demand for the intervention seemed lowest in schools which were both faith-based (RC) and high academic achieving schools. The recruitment rate for these schools was 13% compared to 38% overall. Reasons offered by faith-based and high academic achieving schools for declining to participate were similar to those from other schools: no space in curriculum and current involvement in other research.

**Table 2:** Schools recruited to feasibility trial (June, July, Sept. and Oct. 2014)

School ID	Location	School management type	All pupils eligible free school meals	% Religion per school	Trial allocation
1	Urban	Integrated secondary in deprived area	57.4%	50.4 P 31.3 C 18.3 0	Intervention
2	Semi-urban	Roman Catholic secondary	45.5%	P^a^ C^a^ 0.0 O	Intervention
3	Urban	State grammar	4%	62.7 P 12.4 C 25.0 O	Intervention
4	Urban	State secondary	35.1%	P^a^ C^a^ 16.5 O	Intervention
5	Rural	Roman Catholic secondary in deprived area	48%	0.0 P C^a^ O^a^	Control
6	Semi-urban	Roman Catholic grammar	8.5%	0.0 P 99.5 C O^a^	Control
7	Urban	State grammar	15%	62.7 P 6.5 C 30.5 O	Control
8	Semi-Urban	Integrated secondary	32.4%	74.4 P 5.9 C 19.8 O	Control

*P* Protestant, *C* Catholic, *O* other, *Urban* city, *semi-urban* town in a rural area

^a^Figure concealed under rules of disclosure

**Fig. 1:**
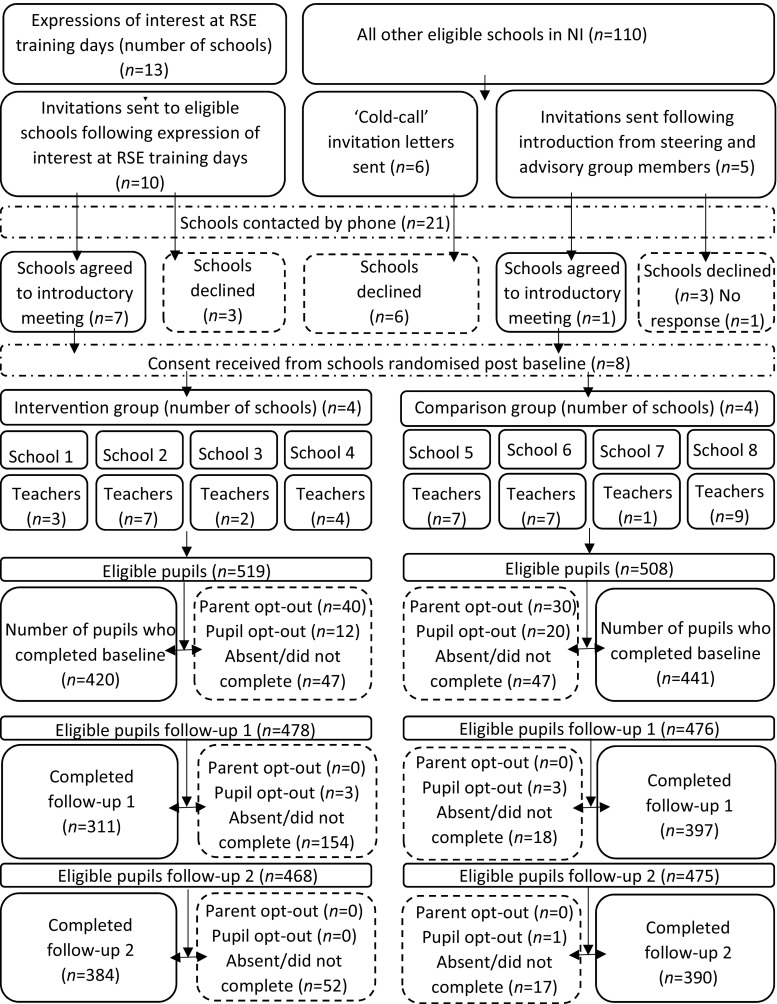
Consolidated Standards of Reporting Trials flow diagram

Within schools, demand was judged by pupil and parental consent to participate. Pupil recruitment rate was acceptable at 80.9%. Parental withdrawal of consent accounted for 6.8% of loss (*n* = 70) and pupil opt-out for 3.1% (*n* = 32). Pupil absence or unavailability at baseline (with absentee questionnaires not returned to the research team) accounted for the remaining 9.1% (*n* = 94). Overall, the study comprised 831 pupils (398 female, 433 male) with 420 pupils randomly allocated to the intervention group and 411 to the control group (Fig. 1).

### Acceptability

Acceptability was first judged by retention in the year-long trial. No school dropped out and pupil withdrawal from the study was low at 10%. The very favourable remarks by teachers and principals across all participating schools showed that the intervention was acceptable.

I thought it was really thought-provoking and, you know, it was realistic. I think that’s the key to something like this. It has to be on their level. I think it’s actually a very good resource... my class would be a class that sometimes would be ‘urgh’…and all that, but they did, they were interested in it and they did have some discussions on it, and to have a discussion with my class is an achievement in itself so (Female teacher, S5).

Pupils were largely very favourable towards the intervention, signalling that it was relatable:

You sometimes get stuff, like resources, and they’re really, like, they’re set by people who don’t relate it very well to people our age and it’s hard to kind of understand. This one was a lot better because it was set with someone our age, like at the present time, doing the same stuff as we’d do. (Male pupil, S7, FG1)

You immediately sort of put yourself in their shoes. (Male pupil, S3, FG1)

They remarked that it was thought provoking in relation to gender norms and got them talking:

Yeah, it was good to see … because, usually, you focus on the girl, like how it would affect the girl, but there, you see…it isn’t affecting the guy physically at all. But it’s…like the moral implications of it, so how he’d think, what would be right, and usually you don’t think of that. Usually, guys are portrayed as just leaving. (Male pupil, S7, FG1)

It’s kind of hard to get a serious conversation between your friends because, a lot of the time, especially … with guys, you know. I think everyone thinks about it but nobody really … Like nobody thinks it’s a suitable topic to discuss within a group of friends. (Male pupil, S7, FG1).

Young people thought that it was useful in helping them to plan not to have a teenage pregnancy:

P1: It helped us learn a bit more about protection and stuff like that there.

P2: Yeah, it was beneficial. Like it reminded me of the dangers of having sex unprotected anyway. (Male pupils, S5, FG2)

… what it’s trying to show you is, like, this can happen to anybody, and like, it makes you think about it more before you, like, maybe do something you’d regret. (Female pupil, S3, FG2).

Nonetheless, there were also some criticisms of the intervention which will inform suggested changes. The IVD, it was thought, would date quickly, if it had not already done so: ‘Look at that phone!’ (Male principal, S7). The most common complaint about the IVD, however, was that there were too many interactive questions and too many demands on the viewer to answer questions when they wanted to let the film flow. Occasionally also, there was concern expressed that there was a disproportionate emphasis on boys’ perspectives in the IVD:

It would be better if you could, like, go from, like, each side, each perspective, if they took, like, the girl and the boy. It would be better because you’d get a rounded perspective of what it would be like. (Male pupil, S7, FG1).

### Implementation/Practicality and Integration

Full attendance was recorded at teacher training sessions offered by the same person within each school. Clear lesson plans and materials enhanced implementation and practicality for teachers within the classroom.

T8: I think we appreciated it from the point of view that we had lessons to follow, timings were there, suggested activities, but also the worksheets. It was great that we were able to get them run-off [photocopied].… So the off-the-shelf materials are great to have. (Female teacher, S4).

However, implementation logs and follow-up interviews with teachers demonstrated that not every teacher followed ‘the wee book’. While all four intervention schools attempted to watch the online version of the film on individual computers with at least one class group, technical difficulties relating to the school network meant that all but one school showed the film on an overhead projector with pupils provided with a paper copy of the questions. Teachers saw the advantages of using individual computers, but most agreed that showing the film on an overhead projector was easier to organise and had benefits for pupils with reading difficulties. Pupils had mixed views and seemed to tolerate both approaches.

P1: Yeah, I liked it on the overhead better…Miss explained it more, on the questions. (Female pupil, S5, FG1)

Overall, interviews with teachers and pupils and observations made by the researchers suggested that teachers wanted to ‘tweak’ the intervention to help it fit with lesson timings and to respond to what engaged the pupils most or caused least disruption. Somewhat ironically, there was a perception by some teachers and female pupils that boys in the classroom were sometimes the hardest to engage and that this disrupted implementation.

P1: No, the boys like… They were so immature about it.

P2: Because the boys were being quite immature so we didn’t get to do it as much. And then the teacher kept stopping to shut them up… (Female pupils, S3, FG2)

Turning to future integration into the RSE school curriculum, in general, teachers and pupils appeared very positive about the potential for this. There was very little variation across the intervention schools in this regard, regardless of management type, faith-based or not.

R: Would you use the resource again?

T2: Yeah, definitely, and it’s a topic that, as I say, the majority of the class were all very interested in. And I said to them, you know ‘If we were to do more things like this, you know, would you be interested?’ and they all put their hand up and said yes, you know, because it’s a topic that’s so current. (Female teachers, S5)

### Results from the Transferability Study

The results from the transferability study endorse the overall acceptability of the intervention within schools, with strong appreciation of the uniqueness of focussing on male perspectives and opportunities afforded to explore the emotions associated with teenage pregnancy by both male and female pupils and teachers.

it shows they [boys] go through the same things we go through. (Wales, S1, f)

However, two stand-out additional lessons were learned which would serve to enhance acceptability in the other nations. The first related to the value of generating a culturally sensitive IVD for England and Wales, where the NI accent of the characters in the original IVD impeded easy understanding, although the accent translated well among Scottish pupils. The second lesson related to the need for greater ethnic diversity among the characters in the IVD which were ‘too white’. This was especially relevant in the London area schools.

### Cost Analysis

The total cost to deliver the intervention was estimated at £5101–, equating to  £1275 per school and £13.76 per pupil (Table 3). The identification of potential recurring resources prior to implementing the intervention allowed the data to be collected prospectively, maximising accuracy of the estimates. Qualitative exploration of the opportunity costs of delivering the intervention indicated that use of this intervention saved teachers’ time which would otherwise have been spent preparing for, or delivering, lessons within the same curriculum.

**Table 3:** Total costs for intervention delivery

Item	Cost (£)
Stage 1 planning and preparation for delivery	
Materials	244.15
Training	565.19
Stage 1:total^a^	809.34
Stage 2: delivery total^b^	4292.34
Overall total^c^	5101.68
Mean cost per teacher (SE)^d^	364.41 (75.24)
Mean cost per school (SE)^d^	1275.42 (341.32)
Mean cost per pupil (SE)^d^	13.66 (4.51)

^a^Based on 16 teachers trained and 420 intervention pupils at baseline

^b^Based on 14 teachers who delivered the intervention

^c^For four schools

^d^SE presented and adjusted for school as the cluster

### Preliminary Effectiveness

Table 4 shows that approximately 3% of the sample reported unprotected sex at baseline (3.6% intervention and 2.5% control) and approximately 5% reported unprotected sex at follow-up, 9 months later (5.4% intervention, 5.6% control). This equates to a 50% increase in unprotected sex in the intervention group (3.6% rising to 5.4%) compared to a more than doubling of unprotected sex in the control group (2.5% rising to 5.6%). The effects were stronger at shorter term follow-up of 5 months with a between-group difference of 2.6%. Nonetheless, the between-group difference in the incidence of unprotected sex of 1.3% (95% CI 0.5 to 2.2%) by 9 months is consistent with effect sizes seen in the literature (Chin et al. 2012) and demonstrates that such an effect size is plausible for this intervention. A difference of 1.4% among this age group has been shown to have a meaningful impact on teenage pregnancy rates (Chin et al. 2012; Henderson et al. 2007; Parkes et al. 2009). However, due attention should also be given to differentials in outcomes for males and females. In the sub-group analysis of the primary outcome measure by sex (Table 4), there was a decrease in percentage of unprotected sex for males in the intervention group (0.5% decrease at FU2), but an increase in females in the intervention arm was evident (4.4% increase at FU2). Thus, the overall reduction in unprotected sex is positive, although the impact may potentially be stronger on males’ behaviour than females’. This is important as in NI males are more likely than females to have had sex before age 16 (Schubotz 2011).

**Table 4:** Primary outcome measure—unprotected sex

Outcome measure	Intervention baseline *n* = 411		Intervention 9 months *n* = 370		Control baseline *n* = 392		Control 9 months *n* = 375		Between-group difference (control/intervention)
Unprotected sex	3.6% (1.8–5.5%)		5.4% (3.1–7.7%)		2.5% (1.0–4.1%)		5.6% (3.3–7.9%)		
Change from baseline			+ 1.8%				+ 3.1%		1.3%
Unprotected sex (stratified by sex)	Male (*n* = 225) 5.3% (2.4–8.3%)	Female (*n* = 186) 1.6% (0.0–3.4%)	Male (*n* = 188) 4.8% (1.7–7.8%)	Female (*n* = 182) 6.0% (2.6–9.5%)	Male (*n* = 191) 4.2% (1.3–7.0%)	Female (*n* = 201) 1.0% (0.0–2.4%)	Male (*n* = 185) 5.4% (2.1–8.7%)	Female (*n* = 190) 5.8% (2.5–9.1%)	
Change from Baseline			− 0.5%	+ 4.4%			+ 1.2%	+ 4.8%	

## Discussion and Conclusions

Teenage pregnancy is a world-wide public health concern and teenage men have a vital yet neglected role to play in prevention (UN 2013; WHO 2017; WHO 2011). Overall, the unique contribution of our study is the demonstration through a cRCT that an RSE intervention targeted to engage teenage men alongside teenage women to think through the gendered responsibilities of preventing unintended teenage pregnacy is acceptable and feasible to implement in mixed-sex schools, with minor recommended changes to enhance overall implementation. The study builds on extant expertise of what makes good RSE interventions but adds an additional component, a gender-sensitive approach to target teenage  men and a gender-transformative approach by encouraging teenage men to share reproductive responsibility. The study thus responds to world-wide policy calls to test interventions designed to include men in the achievement of reproductive health goals. The cost of delivery per pupil in the UK was £13.66, and preliminary results regarding behaviour change were positive. The results showed a reduction in unprotected sex in teenage populations considered to have meaningful impact on teenage pregnancy rates (Chin et al. 2012; Henderson et al. 2007; Parkes et al. 2009).

### Limitations

Some caveats to the interpretation of the results are that the sampling method was a maximum variation quota sample of schools in NI rather than a random sample and broader generalisability is not claimed. The use of the surrogate measure of unprotected sex rather than a biological measure (such as conception rates) introduces self-report bias (Mason-Jones et al. 2016), but veracity of this measure is enhanced by privacy of exam like conditions for data collection and assurances of anonymity and confidentiality of data (Oringanje et al. 2016). Teacher presence at the top of the room helps to maintain exam-like conditions, while visibility of fieldworkers inserting each completed questionnaire into a matched envelope, held in their possession only, helps to authenticate codes of confidentiality. There was loss to follow-up at time 1 due to school absences (19%) with much less at time 2 (11%) for which the main results are reported. Time 1 coincided with end of school term, and lessons were learned about recruitment and retention in school trials (Aventin et al. 2016). Estimates of student attrition will also inform sample size calculations for an effectiveness trial.

### Implications for further research

The results of this feasibility trial demonstrate that a larger effectiveness trial of the intervention is warranted and has now begun (ISRCTN99459996). More broadly, the study will incentivise further trials of RSE interventions targeting adolescent men in the prevention of unintended pregnancy and the greater inclusion of men in addressing the UN’s (2015) Sustainable Development reproductive goals as well as the broader gender equality goals. The fact that this cRCT study also demonstrated that it was possible to include RC faith-based schools in a trial of an RSE intervention encouraging a comprehensive approach to RSE (i.e. both delaying sexual intercourse and consistent use of contraception if sexually active) is also significant. Given the influence of religion in preventing contraceptive use in parts of the world where the need to tackle teenage pregnancy is greatest (for example in Sub-Saharan Africa) (WHO 2017), it is important to recognise that RSE interventions need to be evaluated in settings that are perceived as less receptive.

Evidence supporting the use of interactive digital media in RSE interventions is growing (Bailey et al. 2015). Our paper adds to this evidence and further suggests that it was the combination of the interactive digital medium with an ethnographically informed culturally contextualised IVD (script and actors) that captured the engagement of our target audience. While this was a teacher-delivered intervention, the IVD served to situate the intervention as coming from the voices of young people. Undoubtedly, the culturally sensitive approach will add to the expense of transferring this intervention to other places. Indeed, our transferability study showed that ideally, two different versions of the IVD using appropriate accents are required for use in the UK, with care taken to include greater ethnic diversity where geographically relevant. However, studies replicating our ethnographically informed and culturally sensitive approach to the development of an IVD for inclusion in health promotion programmes, such as marijuana usage (University of Victoria 2013) and smoking cessation (Bottorff et al. 2015) add to the evidence that this approach is worth the investment in generating target audience engagement.

## Electronic supplementary material

ESM 1 (DOCX 16 kb)

## References

[CR1] Acharya DR, Bhattaria R, Poobalan AS, Van Teijlingen E, Chapman GN (2010). Factors associated with teenage pregnancy in South Asia: A systematic review. Health Science Journal.

[CR2] Aventin Á, Lohan M, O'Halloran P, Henderson M (2015). Design and development of a film-based intervention about teenage men and unintended pregnancy: Applying the Medical Research Council framework in practice. Evaluation and Program Planning.

[CR3] Aventin Á, Maguire L, Clarke M, Lohan M (2016). Recruiting schools, adolescents and parents to a sexual-health trial: Experiences, challenges and lessons learned from the Jack trial (NCT02092480). Trials.

[CR4] Bailey Julia, Mann Sue, Wayal Sonali, Hunter Rachael, Free Caroline, Abraham Charles, Murray Elizabeth (2015). Sexual health promotion for young people delivered via digital media: a scoping review. Public Health Research.

[CR5] Bailey JV, Murray E, Rait G, Mercer CH, Morris RW, Peacock R, Nazareth I (2010). Interactive computer-based interventions for sexual health promotion. Cochrane Database of Systematic Reviews.

[CR6] Bottorff JL, Sarbit G, Oliffe JL, Kelly MT, Lohan M, Stolp S, Sharp P (2015). “If I were Nick”: Men’s responses to an interactive video Drama series to support smoking cessation. Journal of Medical Internet Research.

[CR7] Bowen DJ, Kreuter M, Spring B, Cofta-Woerpel L, Linnan L, Weiner D (2009). How we design feasibility studies. American Journal of Preventive Medicine.

[CR8] Braun V, Clarke V (2006). Using thematic analysis in psychology. Qualitative Research in Psychology.

[CR9] Campbell MK, Piaggio G, Elbourne DR, Altman DG, Group, C (2012). Consort 2010 statement: Extension to cluster randomised trials. BMJ.

[CR10] Chin HB, Sipe TA, Elder R, Mercer SL, Chattopadhyay SK, Jacob V (2012). The effectiveness of group-based comprehensive risk-reduction and abstinence education interventions to prevent or reduce the risk of adolescent pregnancy, HIV, and sexually transmitted infections: Two systematic reviews for the guide to community preventive services. American Journal of Preventive Medicine.

[CR11] Connell R (2012). Gender, health and theory: Conceptualizing the issue, in local and world perspective. Social Science & Medicine.

[CR12] Gavin LE, Catalano RF, David-Ferdon C, Gloppen KM, Markham CM (2010). A review of positive youth development programs that promote adolescent sexual and reproductive health. Journal of Adolescent Health.

[CR13] Goesling B, Oberlander S, Trivits L (2017). High-stakes systematic reviews: A case study from the field of teen pregnancy prevention. Evaluation Review.

[CR14] Gruchow HW, Brown RK (2011). Evaluation of the wise guys male responsibility curriculum: Participant-control comparisons. Journal of School Health.

[CR15] Harden A, Brunton G, Fletcher A, Oakley A (2009). Teenage pregnancy and social disadvantage: Systematic review integrating controlled trials and qualitative studies. BMJ.

[CR16] Henderson M, Wight D, Nixon C, Hart G (2010). Retaining young people in a longitudinal sexual health survey: A trial of strategies to maintain participation. BMC Medical Research Methodology.

[CR17] Henderson M, Wight D, Raab GM, Abraham C, Parkes A, Scott S, Hart G (2007). Impact of a theoretically based sex education programme (SHARE) delivered by teachers on NHS registered conceptions and terminations: Final results of cluster randomised trial. BMJ.

[CR18] Herrman JW, Moore C, Rahmer B (2016). Focus on teen men: Evaluating the effectiveness of the wise guys program. Journal of Child and Adolescent Psychiatric Nursing.

[CR19] Hoffmann TC, Glasziou PP, Boutron I, Milne R, Perera R, Moher D, Michie S (2014). Better reporting of interventions: Template for intervention description and replication (TIDieR) checklist and guide. BMJ.

[CR20] Hyde A, Howlett E, Brady D, Drennan J (2005). The focus group method: Insights from focus group interviews on sexual health with adolescents. Social Science & Medicine.

[CR21] Imamura M, Tucker J, Hannaford P, Da Silva MO, Astin M, Wyness L, Temmerman M (2007). Factors associated with teenage pregnancy in the European Union countries: A systematic review. European Journal of Public Health.

[CR22] Lohan M (2015). Advancing research on men and reproduction. International Journal of Men’s Health.

[CR23] Lohan M, Aventin Á, Maguire L, Clarke M, Linden M, McDaid L (2014). Feasibility trial of a film-based educational intervention for increasing boy’s and girl’s intentions to avoid teenage pregnancy: Study protocol. International Journal of Educational Research.

[CR24] Lohan M, Cruise S, O’Halloran P, Alderdice F, Hyde A (2010). Adolescent men’s attitudes in relation to pregnancy and pregnancy outcomes: A systematic review of the literature from 1980–2009. Journal of Adolescent Health.

[CR25] Macdowall W, Jones KG, Tanton C, Clifton S, Copas AJ, Mercer CH (2015). Associations between source of information about sex and sexual health outcomes in Britain: Findings from the third National Survey of sexual attitudes and lifestyles (Natsal-3). BMJ Open.

[CR26] Mason-Jones AJ, Sinclair D, Mathews C, Kagee A, Hillman A, Lombard C (2016). School-based interventions for preventing HIV, sexually transmitted infections, and pregnancy in adolescents. Cochrane Database of Systematic Reviews.

[CR27] Medical Research Council (2006). Developing and evaluating complex interventions: New guidance. https://www.mrc.ac.uk/documents/pdf/complex-interventions-guidance/.

[CR28] Oringanje C, Meremikwu MM, Eko H, Esu E, Meremikwu A, Ehiri JE (2016). Interventions for preventing unintended pregnancies among adolescents. Cochrane Database of Systematic Reviews.

[CR29] Parkes A, Wight D, Henderson M, Stephenson J, Strange V (2009). Contraceptive method at first sexual intercourse and subsequent pregnancy risk: Findings from a secondary analysis of 16-year-old girls from the RIPPLE and SHARE studies. Journal of Adolescent Health.

[CR30] Pound P, Langford R, Campbell R (2016). What do young people think about their school-based sex and relationship education? A qualitative synthesis of young people’s views and experiences. BMJ Open.

[CR31] Pulerwitz J, Hui W, Arney J, Scott LM (2015). Changing gender norms and reducing HIV and violence risk among workers and students in China. Journal of Health Communication.

[CR32] Ritzwoller DP, Sukhanova A, Gaglio B, Glasgow RE (2009). Costing behavioral interventions: A practical guide to enhance translation. Annals of Behavioral Medicine.

[CR33] Santelli JS, Lindberg LD, Finer LB, Singh S (2007). Explaining recent declines in adolescent pregnancy in the United States: The contribution of abstinence and improved contraceptive use. American Journal of Public Health.

[CR34] Schubotz, D. (2011). Messed up? Sexual lifestyles of 16 year olds in Northern Ireland. ARK.

[CR35] Stephenson JM, Oakley A, Johnson AM, Forrest S, Strange V, Charleston S (2003). A school-based randomized controlled trial of peer-led sex education in England. Controlled Clinical Trials.

[CR36] Swann C, Bowe K, McCormick G, Kosmin M (2003). Teenage pregnancy and parenthood: A review of reviews. Evidence briefing for health development agency.

[CR37] United Nations (2015). Sustainable Development Goals. https://sustainabledevelopment.un.org/content/documents/21252030%20Agenda%20for%20Sustainable%20Development%20web.pdf.

[CR38] University of Victoria (2013). Cycles - An Educational Resource Exploring Decision Making and Marijuana Use among Young People. https://www.uvic.ca/research/centres/carbc/publications/helping-schools/cycles/index.php.

[CR39] Wang S, Moss JR, Hiller JE (2005). Applicability and transferability of interventions in evidence-based public health. Health Promotion International.

[CR40] WHO (2011). *Evidence for gender responsive actions to prevent and manage adolescent pregnancy*.http://www.euro.who.int/__data/assets/pdf_file/0008/158093/316637_WHO_brochure_226x226_5-AdolecentPregnancy.pdf.

[CR41] WHO (2017). *Maternal, newborn, child and adolescent health*. http://www.who.int/maternal_child_adolescent/en/.

