# Supplementary material for: Can Teenage Men Be Targeted to Prevent Teenage Pregnancy? A Feasibility Cluster Randomised Controlled Intervention Trial in Schools
Source: Prev Sci. 2018 Jul 18;19(8):1079–90. doi: 10.1007/s11121-018-0928-z (PMC6208578; doi:10.1007/s11121-018-0928-z)
Supplement: Supplementary file 1 — (DOCX 16 kb) [file 11121_2018_928_MOESM1_ESM.docx]

**PRIMARY OUTCOME**

The surrogate measures (alternatives to the use of teenage pregnancy) used in this feasibility study to create the composite primary outcome measure were abstinence from sexual intercourse (delay initiation of sex or return to abstinence) or avoidance of unprotected sexual intercourse (consistent correct use of contraception that does not include withdrawal or natural family planning). In this trial we used a number of questionnaire items to obtain this outcome (see below).

At baseline, follow-up one and two, the delay of initiation of sex was determined using the following questionnaire item.

- Have you ever experienced penetrative sex (penis-vagina) with another person?
  - Yes
  - No

At follow-up one data collection a return to abstinence was determined using the following questionnaire item. If pupils responded more than five* months ago, they had abstained since baseline.

- When was the last time you had penetrative sex with someone?
  - Less than a week ago
  - More than a week but less than a month ago
  - 1-4 months ago
  - More than 5 months ago *(when baseline data collection occurred)*

At follow-up two data collection a return to abstinence was determined using the following questionnaire item. If pupils responded not since June 2015, they had abstained in the last four months.

- How many times have you had penetrative sex since you completed the last questionnaire in June 2015? *(which was four months prior)*
  - Not since June 2015
  - Once
  - Between two and five times
  - Between six and ten times
  - Between 11 and 20 times
  - More than 20 times

*In some schools follow-up one took place six months post-baseline. If this was the case the question was changed to reflect this.

Avoidance of unprotected sexual intercourse (consistent correct use of contraception that does not include withdrawal or natural family planning) was determined using the following two questionnaire items.

- Thinking about ALL the times you have had sex EVER. How often were you or your partner protected against pregnancy? (Do not count the withdrawal method or emergency contraception – “the morning after pill”).
  - Never
  - Not very often
  - About half the time
  - Most of the time
  - Always
- The last time you had penetrative sex, did you or your partner use any form of contraception?
  - None for me, don’t know about partner
  - None for either of us
  - Yes

If yes, please tick all the contraceptives that you used the last time you had sex: Type:

- Pill, patch or vaginal ring
- Condoms
- Emergency contraceptive pill
- Injection
- Implant
- Withdrawal method
- Intrauterine device
- Diaphragm/Cap/Spermicide
- Natural family planning
- Don’t know name
- Other
